# Variation in blubber cortisol levels in a recovering humpback whale population inhabiting a rapidly changing environment

**DOI:** 10.1038/s41598-022-24704-6

**Published:** 2022-11-24

**Authors:** L. J. Pallin, N. Botero-Acosta, D. Steel, C. S. Baker, C. Casey, D. P. Costa, J. A. Goldbogen, D. W. Johnston, N. M. Kellar, M. Modest, R. Nichols, D. Roberts, M. Roberts, O. Savenko, A. S. Friedlaender

**Affiliations:** 1 Fundación Macuáticos Colombia, Calle 27 # 79-167, Medellín, Colombia; 2 Programa Antártico Colombiano, Avenida Ciudad de Cali #51 - 66, Oficina 306, Edificio World Business Center – WBC, Bogotá, D.C. Colombia; 3 Marine Mammal Institute, Department of Fisheries, Wildlife and Conservation Sciences, Oregon State University, Hatfield Marine Science Center, 2030 SE Marine Science Drive, Newport, OR 97365 USA; 4 Institute for Marine Science, University of California Santa Cruz, Ocean Health Building, 115 McAllister Way, Santa Cruz, CA 95060 USA; 5 California Ocean Alliance, 9099 Soquel Ave, Aptos, CA 95003 USA; 6 Department of Biology, Hopkins Marine Station, Stanford University, 120 Ocean View Blvd, Pacific Grove, CA 93950 USA; 7 Division of Marine Science and Conservation, Nicholas School of the Environment, Duke University Marine Laboratory, 135 Duke Marine Lab Road, Beaufort, NC 28516 USA; 8 Marine Mammal Turtle Division, Southwest Fisheries Science Center, National Marine Fisheries Service, National Oceanic and Atmospheric Administration, 8901 La Jolla Shores Drive, La Jolla, CA 92037 USA; 9 Department of Ocean Sciences, University of California Santa Cruz, Ocean Health Building, 115 McAllister Way, Santa Cruz, CA 95060 USA; 10 National Antarctic Scientific Center of Ukraine, 16 Taras Shevchenko Blvd., Kyiv, 01601 Ukraine; 11 Ukrainian Scientific Center of Ecology of the Sea, 89 Frantsuzsky Blvd., Odesa, 65009 Ukraine; 12 Present Address: Department of Ecology and Evolutionary Biology, University of California Santa Cruz, Ocean Health Building, 115 McAllister Way, Santa Cruz, CA 95060 USA

**Keywords:** Steroid hormones, Animal physiology, Climate-change ecology, Conservation biology, Population dynamics

## Abstract

Glucocorticoids are regularly used as biomarkers of relative health for individuals and populations. Around the Western Antarctic Peninsula (WAP), baleen whales have and continue to experience threats, including commercial harvest, prey limitations and habitat change driven by rapid warming, and increased human presence via ecotourism. Here, we measured demographic variation and differences across the foraging season in blubber cortisol levels of humpback whales (*Megaptera novaeangliae*) over two years around the WAP. Cortisol concentrations were determined from 305 biopsy samples of unique individuals. We found no significant difference in the cortisol concentration between male and female whales. However, we observed significant differences across demographic groups of females and a significant decrease in the population across the feeding season. We also assessed whether COVID-19-related reductions in tourism in 2021 along the WAP correlated with lower cortisol levels across the population. The decline in vessel presence in 2021 was associated with a significant decrease in humpback whale blubber cortisol concentrations at the population level. Our findings provide critical contextual data on how these hormones vary naturally in a population over time, show direct associations between cortisol levels and human presence, and will enable comparisons among species experiencing different levels of human disturbance.

## Introduction

Wildlife biologists and natural resource managers must identify at-risk individuals and populations and determine why they are threatened. The underlying biological mechanisms associated with these threats are often poorly understood. Increasingly, the physiological response of wildlife to ecological perturbations is used as an indicator of poor population health^1,2^, and the associated costs (e.g., immunosuppression) and mechanisms of these stressors are of particular importance. Physiological indicators provide a directly integrated measure of the response in behavior and physiology and how these manifest to impacts of individual fitness and changes in population dynamics over time^1,3,4^.

Glucocorticoids (GC) are a group of corticosteroid hormones produced in the adrenal cortex that mainly regulate metabolism and endocrine responses to stressors^5^. For example, GC concentrations primarily suppress glucose uptake, providing energy to the body when an organism’s energy demands exceed energy availability (e.g., fight or flight response)^6^. However, it is important to note that GC physiology can be both species- and context-specific, and thus careful consideration needs to be used when interpreting values^6^.

In vertebrates, GCs (including cortisol) have been analyzed in several different sample matrices as possible indicators of a stress state. In the last 20 years, captive marine mammal research programs, in which endocrine measures can be easily obtained, have enhanced our understanding of stress responses to acute stimuli^7,8^. How these short-term responses translate into long-term population impacts, particularly in large, wild, long-lived vertebrates, remains poorly understood^9,10^. Thus, it is vital to assess the potential effects that repeated exposure to stressors may have on population dynamics, particularly those experiencing rapid changes in human activities and most susceptible to current and projected climatic changes. If we measure GCs in tissues that reflect long-term exposure to stressors, it is possible to monitor changes in the stress states experienced by populations over prolonged periods^11^.

Stressors in natural populations occur in a variety of forms. Studies on marine mammals have documented associations between cortisol levels and human activity, such as vessel traffic and noise^9,12^. Around the Antarctic Peninsula, baleen whales have and continue to experience a variety of threats ranging from direct depletion from commercial whaling, prey limitations and habitat change driven by rapid warming, and an increase in human activity in the form of ecotourism. Baleen whale populations in the Southern Hemisphere (SH) were severely depleted during the commercial whaling period (1904–1980)^13^. In some regions of the SH, like the nearshore waters along the Western Antarctic Peninsula (WAP), whales are now confronted with some of the most severe impacts of global change. For example, the WAP, an essential feeding ground for an estimated 11,700 humpback whales^14^, has experienced a rise in winter temperature of nearly 5 °C since the 1950s, resulting in the collapse of ice shelves, the retreat of glaciers, and the exposure of new terrestrial habitat^15^. While the lack of other baleen whales in this region has likely reduced overall competition for resources^16^, there is evidence that prey resources are decreasing due to reduced cycling of nutrients^17^, and these reductions in prey are manifesting as decreased pregnancy rates in female humpback whales along the WAP [Pallin et al. in prep]. Further, humpback whales are the most abundant baleen whale species in this region and thus are considered sentinel species for ecosystem processes and climate-related changes. All these ecosystem changes have critical implications for the population health and growth of whales in this region.

Humpback whales along the WAP appear to be recovering quite rapidly^18^, even as human activities (i.e., vessel tourism and the commercial krill fishery) in this region are quickly expanding^19^. Specifically, along the WAP, we have seen a rise in tourism from 6700 visitors among 59 voyages run by 12 vessels in 1990 to over 73,000 visitors among 408 voyages run by 62 vessels during the 2020 season^20^. Previous studies have shown associations between cumulative anthropogenic impacts (i.e., sound pollution, fishing, vessel traffic) and cortisol levels in baleen whales in other parts of the world^12,21^. Therefore, Antarctic whales may be stressed by human activities related to ecotourism^12,22–24^. However, to the best of our knowledge, no studies have assessed this relationship in populations that are recovering largely in the absence of direct human activity.

Despite the International Association of Antarctic Tour Operators (IAATO) having self-imposed regulations on approaching whales and, more recently, on vessel speed, there is no way to account for the cumulative numbers of vessels and time these vessels are spending in the presence of whales. Understanding these impacts is crucial for species like humpback whales that exhibit two distinct seasonal modes. Humpback whales along the WAP migrate annually from their low latitude breeding grounds off the Northwest coast of South America to their high latitude feeding grounds along the WAP^25^. These two distinct behaviors make particular times of their annual life history and demography more susceptible to stressors. In whales in the wild, collecting blood for endocrine monitoring is currently not feasible. Likewise, collection of feces and blow sputum, although non-invasive, is logistically challenging and susceptible to contamination from seawater^26^. Therefore, skin-blubber biopsies, which can be readily collected across both seasonal life-history strategies and archived, provide a viable opportunity to test whether prolonged exposure to potential stressors is related with increased blubber cortisol concentrations.

The COVID-19 pandemic created a unique opportunity by removing humans (e.g., science and tourism) from waters around the WAP in 2021, resulting in an “anthropause.” Quantifying these impacts in the absence of anthropogenic disturbance is often challenging. The goals of this study were to (i) describe the demographic and monthly trends in blubber cortisol levels of humpback whales during the feeding season around the Antarctic Peninsula over two years (2019–2020) when human presence was consistent and (ii) test whether reductions in vessel presence along the WAP in 2021 as a result of the COVID-19 pandemic were associated with a decrease in blubber cortisol levels. Our findings provide critical contextual data on how these hormones vary naturally in a population. Further, our data provide the basis for more focused comparisons with other populations of whales in locations with differing levels of human activity.

## Results

**Figure 1:**
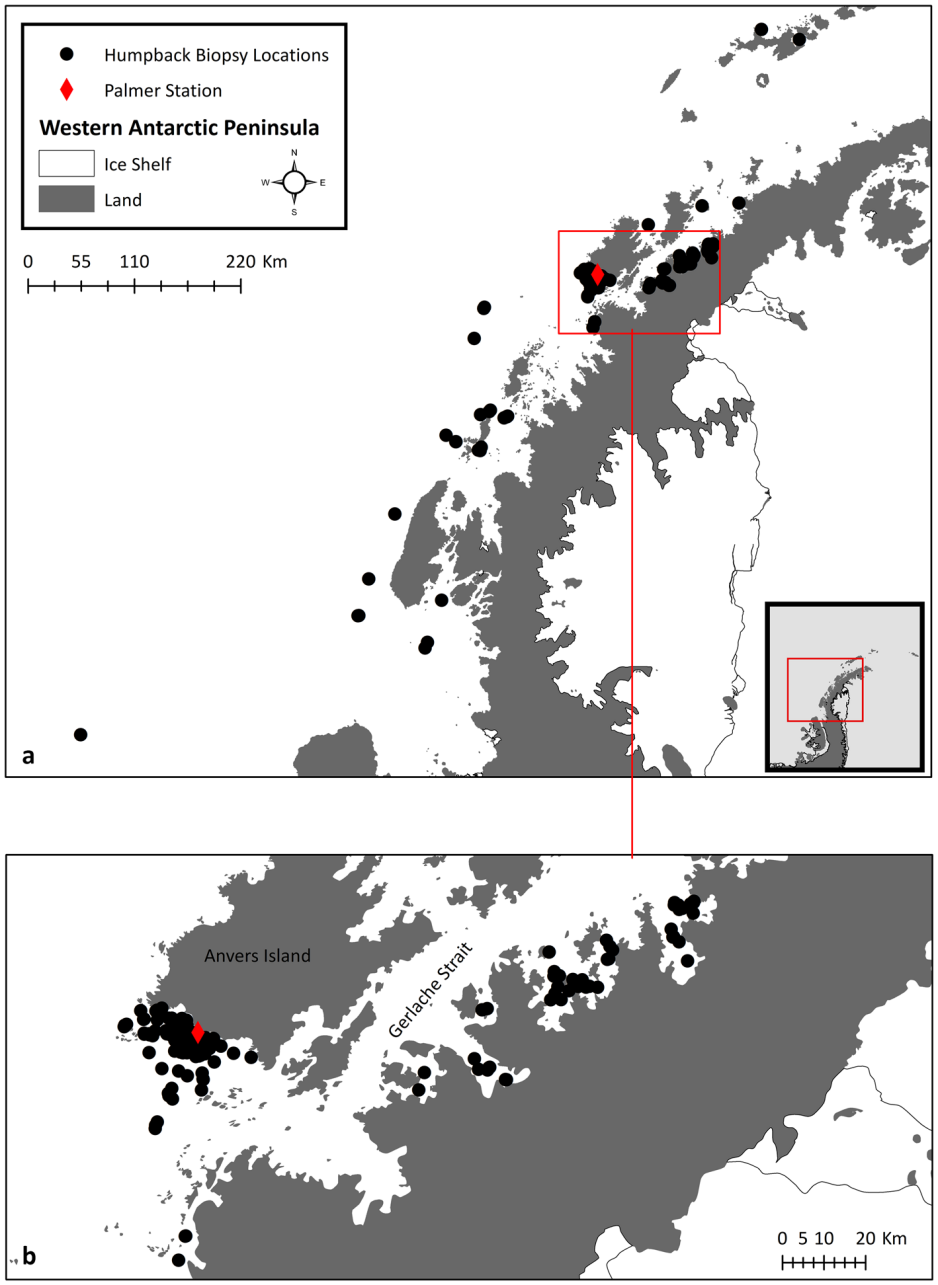
Sampling locations of humpback whales along the Western Antarctic Peninsula (**a**) and in the (**b**) Gerlache Strait and adjacent bays during the 2019–2021 field seasons. Maps were created using ArcMap version 10.8.2^70^ (2022, https://www.esri.com/en-us/home).

We analyzed biopsy samples collected from 305 individual humpback whales in the nearshore waters around the WAP over the course of three field seasons from 2019 to 2021 (2019 n = 134, 2020 n = 145, 2021 n = 26; Fig. 1). Sampling logistics were greatly reduced during 2021 due to the COVID-19 pandemic. Annual sampling details can be found in Table 1. The mean cortisol concentration for the 305 individual whales sampled was 0.4 ± 0.28 ng/g.

**Table 1:** Blubber cortisol levels (mean ± SD ng/g wet weight) in biopsied humpback whales across years and different sex and demographic groups.

Year	Sex	Demographic class	Blubber cortisol (ng/g wet weight)
2019 (n = 134)			0.45 ± 0.34
	M (n = 50)		0.53 ± 0.46
	F (n = 84)		0.4 ± 0.24
		Lactating (n = 15)	0.38 ± 0.27
		Lactating and Pregnant (n = 8)	0.56 ± 0.3
		Pregnant (n = 31)	0.42 ± 0.22
		Resting (n = 30)	0.34 ± 0.22
2020 (n = 145)			0.38 ± 0.22
	M (n = 57)		0.36 ± 0.25
	F (n = 88)		0.39 ± 0.2
		Lactating (n = 7)	0.28 ± 0.12
		Lactating and Pregnant (n = 6)	0.46 ± 0.18
		Pregnant (n = 22)	0.47 ± 0.22
		Resting (n = 53)	0.37 ± 0.19
2021 (n = 26)			0.28 ± 0.14
	M (n = 15)		0.31 ± 0.16
	F (n = 11)		0.25 ± 0.1
		Lactating (n = 3)	0.24 ± 0.09
		Lactating and Pregnant (n = 1)	0.24
		Pregnant (n = 2)	0.23 ± 0.08
		Resting (n = 5)	0.26 ± 0.14

### Individual identification and sex

On average, 9.97 loci were successfully genotyped per individual. The average P_ID_ for any given combination of 7 loci ranged from 8.02 × 10^–11^ to 5.74 × 10^–8^, consistent with previous studies. We analyzed 183 individual females and 122 individual males across all three years (Table 1).

### Validation of humpback cortisol assays

**Figure 2:**
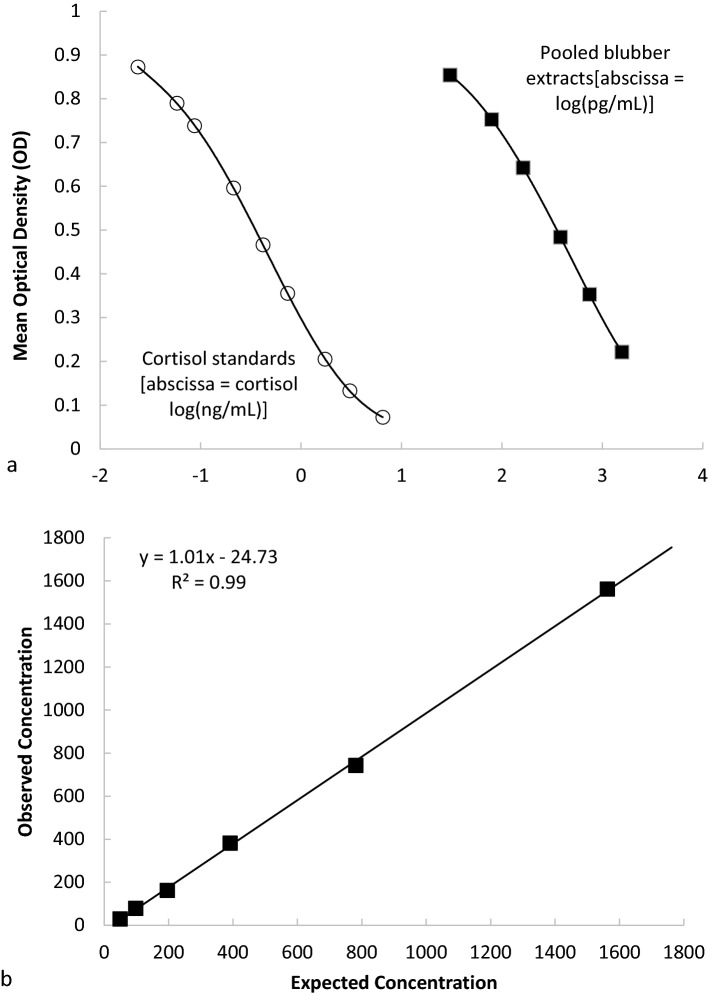
Enzyme immunoassay (EIA) validations for cortisol extracted from blubber biopsy samples in humpback whales. (**a**) Serial dilutions of extracts (shaded squares) showed strong parallelism with the standards of the cortisol EIA (open circles) and good accuracy (**b**) demonstrated by the positive linear relationship of known cortisol concentrations against apparent concentrations in spiked samples (R^2^ = 0.999, slope = 1.01); Both tests indicate that the assay is measuring the same antigens in the blubber as in the standards and therefore suitable for use with humpback whale blubber tissues extracts. Four individual females were represented in the pooled blubber extracts.

Based on the concentrations observed from a series of spiked controls, our average extraction efficiency for the cortisol assay was 82.40% ± 11.75 (minimum 60.54%, maximum 100.14%). The EIA standards and the pooled serially diluted blubber extracts exhibited statistical parallelism and high accuracy (Fig. 2, R^2^ = 0.999, slope = 1.01); an indication that the assay is measuring the same antigens in the blubber as in the standards and is, therefore, suitable for use with humpback whale blubber tissues extracts. Additionally, our calculated intra-assay and inter-assay COV from a series of replicated samples were 6.14 and 14.93%, respectively. These results are consistent with previous studies on humpback whales^10^. Thirty-five samples had values that were undetectable by the assay used in this study.

### Demographic and monthly variation in cortisol concentrations across years

**Figure 3:**
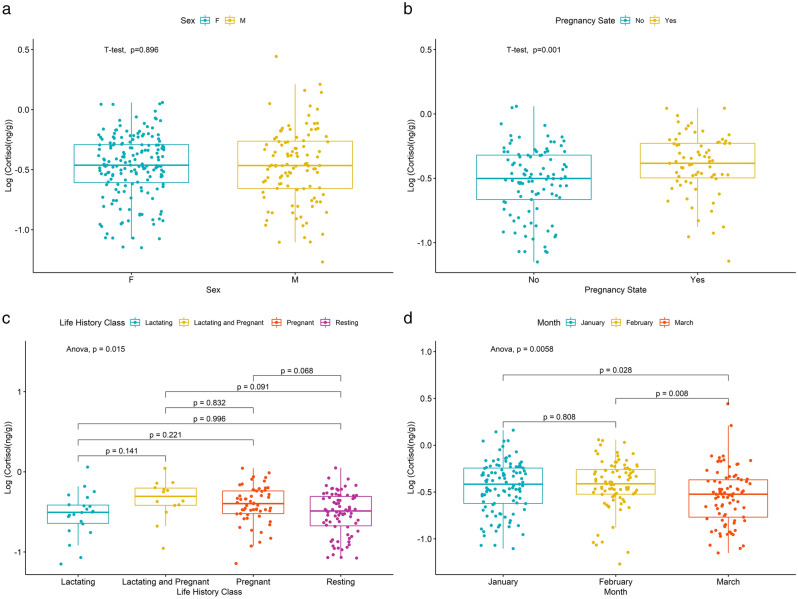
Blubber cortisol concentrations (ng/g wet weight) of humpback whales sampled along the Western Antarctic Peninsula during 2019–20. Blubber cortisol concentrations by sex (**a**), pregnancy state (**b**), life history class (**c**), and across months (**d**).

The mean cortisol concentration for all female and male humpback whales sampled in 2019 and 2020 was 0.40 ± 0.22 and 0.44 ± 0.37 ng/g, respectively (Table 1). These were not significantly different (t = -0.131, df = 196.8, *p* = 0.896, Fig. 3a). Of the 172 individual female humpback whales, 71 were classified as pregnant (41.28%, mean = 0.46 ± 0.22 ng/g) and 101 individuals were classified as not pregnant (mean = 0.36 ± 0.21 ng/g). Pregnant females had significantly higher cortisol levels than not pregnant females (t = − 3.250, df = 161.28, *p* = 0.001, Fig. 3b). Of the 71 pregnant females, 57 females were classified as pregnant but presumably not lactating (mean = 0.44 ± 0.22 ng/g) while 14 were assigned as lactating and pregnant (mean = 0.51 ± 0.25 ng/g). Additionally, of the 101 females classified as not pregnant, 79 females were assigned as resting (mean = 0.36 ± 0.2 ng/g), and 22 were assigned as lactating (mean = 0.35 ± 0.23 ng/g). We observed a significant difference in the cortisol concentrations among these different life history groups (r^2^ = 0.044, F _3168_ = 3.603, *p* = 0.015, Fig. 3c). However, a post hoc analysis revealed no two life history groups were different from each other.

During the 2019 and 2020 season, we sampled 109 humpbacks in January (mean = 0.43 ± 0.27 ng/g), 85 in February (mean = 0.44 ± 0.23 ng/g), and 85 in March (mean = 0.36 ± 0.35 ng/g), and found a significant decrease in blubber cortisol levels across the feeding season (r^2^ = 0.030, F _2,276_ = 5.253, *p* = 0.006, Fig. 3d). A post hoc multiple comparisons analysis revealed that the cortisol concentrations among humpback whales sampled in March (later in the feeding season) were significantly lower than in both January (*p* = 0.028) and February (*p* = 0.008) (earlier in the feeding season). When comparing the relationship between cortisol concentrations and day of year, we found no significant relationship (r^2^ = 0.008, F _1303_ = 2.55, *p* = 0.111).

### Association of population-level stress state and human activity

**Figure 4:**
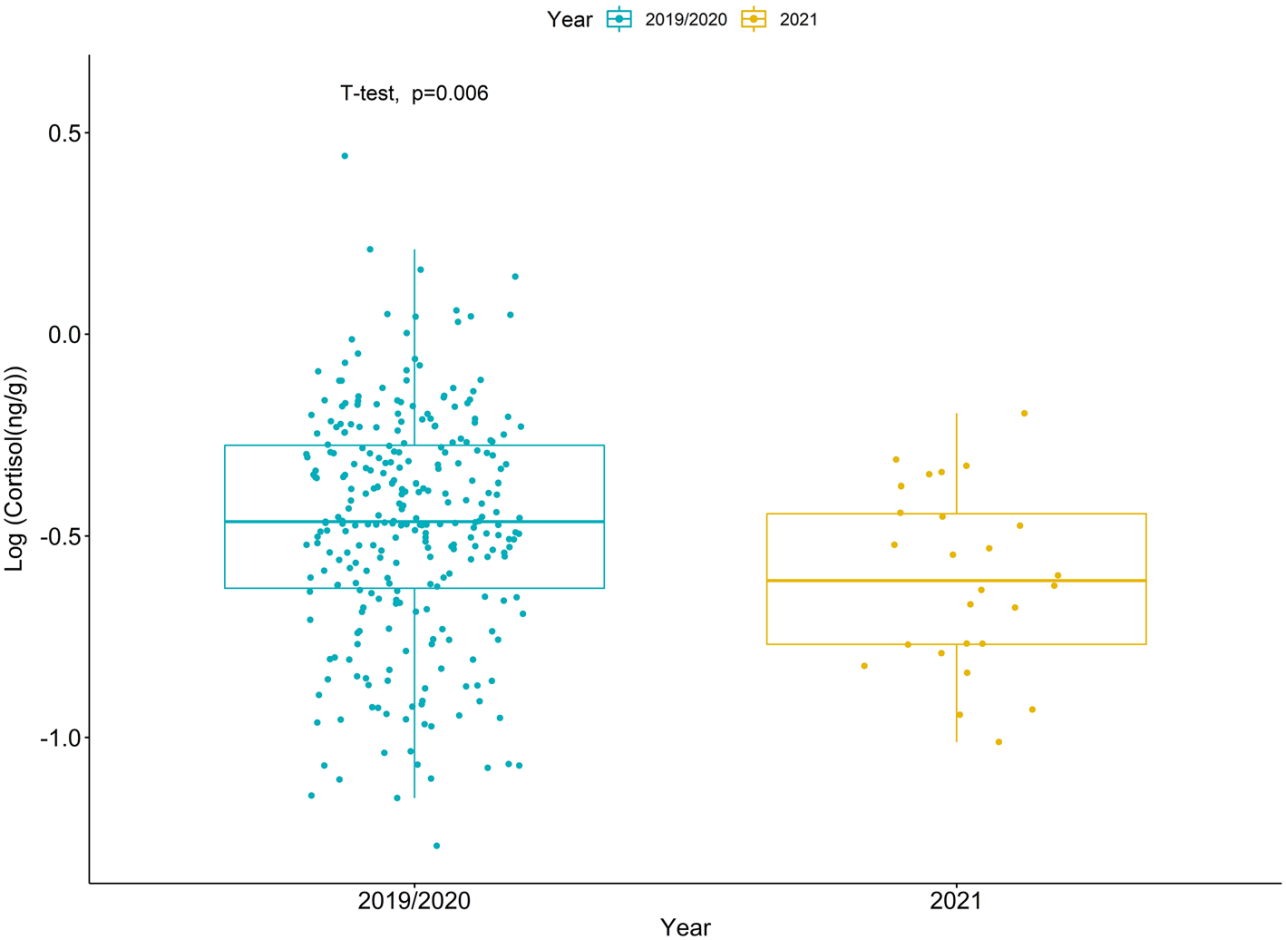
Variation in blubber cortisol concentrations (ng/g wet weight) of humpback whales sampled along the Western Antarctic Peninsula during periods of high (2019/2020) and low (2021) vessel presence. Cortisol concentrations are log-transformed.

The number of IAATO-registered vessels along the WAP was greatly reduced in the 2021 ecotourism season. Specifically, IAATO reported 360 voyages among 56 vessels in 2019, 408 voyages among 62 vessels in 2020, and only two voyages among two registered yachts in 2021. The mean cortisol concentrations for 2019, 2020, and 2021 were 0.45 ± 0.34 ng/g, 0.38 ± 0.22 ng/g, and 0.28 ± 0.14 ng/g respectively. When combined based on similarity in levels of human activity, the mean cortisol concentration during the 2019/2020 seasons (mean = 0.41 ± 0.29 ng/g) was significantly higher than the mean cortisol concentrations during the 2021 season (mean = 0.28 ± 0.14 ng/g; t = 2.913, df = 33.1, *p* = 0.006, Fig. 4).

## Discussion

Our study provides critical contextual data on how blubber cortisol levels vary naturally in a population of humpback whales inhabiting Antarctic waters and shows that a post-COVID-19 decrease in vessel activity along the WAP was related to a decrease in blubber cortisol levels. Cortisol concentrations in humpback whales along the WAP were generally low but comparable to levels measured in the blubber of other baleen whale species^10,27,28^. This information is essential for monitoring population dynamics in the face of continued anthropogenic and environmental changes. Furthermore, these results will facilitate inter-population comparisons of cortisol levels among areas with differing levels of human activity and disturbance.

We observed no significant difference in cortisol concentrations between male and female humpback whales. However, we did observe significant variation between different demographic groups of females. Several studies have found that baseline and stress-induced GC levels vary due to intrinsic biological factors, such as sex, reproductive state, and age in other species^3,29–31^. For example, male North Atlantic right whales show significantly higher levels of cortisol related to the mobilization of energy reserves needed for breeding activity^27,29^. Given that male humpback whales sampled in the current study were sampled roughly four months post peak breeding, we would not anticipate that they would have elevated cortisol levels as a result of previous breeding activities. In our study, pregnant and lactating while pregnant females had the highest observed cortisol levels among female-specific demographic groups, similar to other studies^29,32^. Pregnancy involves a series of metabolic and physiological adjustments to help maintain the increased energy expenditure needed for fetal development^33,34^. For pregnant whales, endocrine responses reported to date include high progesterone concentrations to help establish and maintain pregnancy^18,28,35^ and elevated cortisol^29^. Increased cortisol concentrations in pregnant whales is not unexpected, as the adrenal gland responds to endocrine changes throughout gestation^33^. Conversely, resting and lactating but not pregnant females had the lowest cortisol levels, consistent with other studies^36^.

Many studies of terrestrial mammals have also shown that age drives changes in baseline GC levels^3,31^. In our study, the age of sampled whales is unknown; thus, we cannot account for its potential effect on GC levels. It is also important to note that while we found significant differences in cortisol among different life history groups in humpback whales, similar studies on both humpback^10^ and blue whales^28^ found no effect of life history stage. This could be a result of low sample sizes and/or population-specific differences. Future work should incorporate detailed sighting histories in combination with cortisol monitoring to better understand how susceptible different age and life history groups are to the continued changes occurring along the WAP.

We observed higher cortisol in humpback whales sampled during the early part of the feeding season in January and February than later in March. The WAP is a very dynamic marine ecosystem, and the rapid climatic shifts in this region exacerbate the annual and seasonal variability in the ecosystem^15,37,38^. It is thought that cortisol regulation evolved as a mechanism to balance energetic demands between behavioral modes associated with different seasonal states^3^. In Steller sea lions, cortisol levels significantly increased during periods of nutritional stress and were negatively correlated with percent body fat^31^. Thus, it is likely that temporal variability in abiotic (i.e., available habitat) and biotic (i.e., prey availability) stressors may manifest in changes in population cortisol levels in humpback whales along the WAP.

Steroid hormone integration and clearance in blubber, particularly in large whales, is still poorly understood. It is possible that the higher levels early in the feeding season could be a residual carryover from the most recent breeding season^7,39^. Several species, including North Atlantic right and humpback whales, show increases in baseline cortisol levels in relation to breeding activities^3,10,29^. Conversely, these high early season cortisol levels, followed by a decrease later in the season as thermal stores are likely replenished, could also be the result of homeothermic responses (i.e., increase in baseline metabolic rate) associated with polar waters^34,40,41^. Similar occurrences have been observed in both dolphins and pinnipeds^40,41^. However, thermal models suggest that the lower critical temperature for large whales is much lower than the minimal seawater temperature they encounter^42^. Therefore, the monthly effects documented in this study are more likely nutritionally derived, as in the case of the Steller sea lions^31^. This is also supported by recent observations that humpback whale feeding rates are highest early in the feeding season (Nichols et al.^43^) when whale body condition is at its lowest^44^, and they are nutritionally stressed. While cortisol likely plays a role in the physiological response to nutritional stress and changes in energy stores, other hormones work in tandem to maintain homeostasis. Thus, continued annual and seasonal sampling of this species across breeding and feeding grounds and incorporating other nutritional biomarkers (e.g., triiodothyronine) is needed to better understand how short and long-term environmental variability affects population-level variation in cortisol levels.

While we observed significant differences in cortisol concentrations across the feeding season and among some demographic groups, these differences might not be biologically significant. Other studies have documented much wider ranges in blubber cortisol concentrations in cetaceans. However, these were done in regions that, on average, contain a greater human presence compared to the WAP. For example, Graham et al.^27^ reported a six-fold increase in the concentrations between entangled and healthy North Atlantic right whales. Kellar et al.^45^ found that stranded common dolphins had, on average, 6.1 times higher blubber cortisol values than bycaught dolphins in the eastern North Pacific. These studies clearly documented that high adrenal activation due to stressful events can be captured in blubber tissue. We sampled one juvenile humpback whale (female) during the 2022 (January) field season that was actively entangled in fishing gear around its caudal peduncle and fluke (unpublished data). This individual had a mean blubber cortisol concentration of 0.70 ng/g. Though only 1.75 times greater than our overall mean concentration (0.40 ng/g),and a much smaller difference in magnitude than reported above, this whale was under stress as the entanglement likely occurred outside of the Antarctic, implying the animal had been carrying the gear during its migration and for the entire period it had been on the feeding grounds. If human activity affects cortisol levels in humpback whales along the WAP, the magnitude of this effect appears smaller compared to other studied populations of baleen whales^12^. Furthermore, an individual’s behavioral and endocrine response to a stressor are complex and highly variable^6^. As a result, careful consideration must be taken when directly connecting stress physiology to individual and population level effects on fitness and health. Thus, it is important that humpback whale blubber cortisol levels continue to be monitored. This will be necessary to understand how cortisol variability translates into meaningful biological differences at the population and individual level.

We observed a significant decrease in cortisol concentrations in 2021, a year when human activity along the WAP was greatly reduced due to the COVID-19 pandemic. However, our sample size for the 2021 season is relatively small. Even so, we do not believe these findings result from a temporal artifact in our sampling because the median ordinal dates for which samples were collected across the three seasons (2019–2021) are 36, 46, and 35, respectively. Ecotourism, especially whale watching, is a growing industry worldwide, particularly along the WAP. Vessel disturbance, both from the physical presence of boats and the associated vessel noise, has both short-term and long-term physiological and behavioral impacts on marine mammals, including humpback whales^12,23,46–49^. Sound, like that generated from cruise ships, is a persistent source of low-frequency ocean noise and travels effectively in sea water, moving approximately five times faster than it does in air^50^. These properties allow sound to dramatically alter the soundscape of marine environments, masking essential sounds produced and heard by marine animals, including whales, as well as potentially changing animal behavior and physiology^50^. For example, post-9/11 decreases in background underwater noise from reduced ship traffic corresponded to a decrease in fecal GC levels in North Atlantic right whales^12^. Conversely, in Alaskan humpback whales, cortisol levels in groups of whales in high vessel-use areas were not significantly higher than in other, low vessel regions^9^. A conclusion drawn from this study was that humpback whales in Alaska were likely habituated to vessel presence. Though this study was conducted on a separate population of humpback whales, it is plausible that WAP humpback whales also have some internal habituation to vessel presence. Several studies have documented decreases in cortisol levels as a result of habituation^51^. However, even if habituated, this does not necessarily mean that whales will not become more susceptible to collisions, propeller strikes, and entanglement in fishing gear^52^, especially as the ecotourism and krill fishing industry grow along the WAP. Continued monitoring of this population is required to better understand the interplay between demographic, temporal, and anthropogenic stressors and how these manifest in both short and long-term impacts on population health and dynamics.

While our sample size in 2021 was relatively small, we still detected a significant decrease in cortisol levels when human presence (i.e. number of vessels) along WAP was greatly reduced. Without a planned cessation of ecotourism, future work is needed to further characterize and compare the relationship between human disturbance and blubber cortisol levels in humpback whales given the natural variability in GC levels. However, this study demonstrates the continued efficacy of using blubber biopsies to measure hormone concentrations from whales in the wild and provide critical contextual information on how these hormones vary naturally in a population of recovering baleen whales. While we found an association between cortisol levels and human activity, this population is relatively naïve in its exposure to human activity as there are minimal human activities in these waters outside of tourism (as well as scientific ships and a commercial krill fishery). Throughout their broad distributions in coastal areas in both hemispheres, humpback whales occur in both some of the most heavily trafficked regions and some of the least. This dichotomy makes humpback whales an ideal species to use for comparing how human disturbance impacts cortisol levels among wildlife. Specifically, as the environment continues to change along the WAP and human activities continue to grow in this region, we may observe changes in the stress states of these whales over the long-term. By combining these measurements with new technologies for assessing biomarkers from additional sample matrices (i.e., blow sputum), more specific markers of nutritional state (i.e., thyroid hormones)^31^, as well as photogrammetry to evaluate body condition, we can better understand the relationships between human disturbance and animal stress.

## Methods

### Biopsy collection

We collected skin and blubber biopsy samples from humpback whales during the 2019–2021 austral summer and fall (January-July) field seasons using standard biopsy techniques^53^. Samples were collected opportunistically during dedicated research cruises or from platforms of opportunity, including tour vessels, in the nearshore waters along the western side of the Antarctic Peninsula (Fig. 1). Further, a majority of our samples were collected in the bays and fjords adjacent to the Gerlache Strait and near Palmer Station at the Southern end of Anvers Island. Both of these regions are highly used by humpback whales^54^ and tourism vessels^19^.

We used a crossbow with modified bolts and 40 mm stainless steel cutting tips (CetaDart) to obtain samples from a distance of 10–30 m targeting the area of the body below the dorsal fin when the whale surfaced to breathe^55^. Humpback whales were sampled opportunistically from all age and sex classes, including calves. Samples were stored frozen whole at -20° C until used for analysis. Supplementary data (i.e., location, group size, group composition) were also recorded with every biopsy event.

### DNA profiling

A standard DNA profile, including sex-specific markers, and microsatellite genotypes, was used to identify individual whales. DNA was extracted from the skin-blubber interface using a commercially available kit (DNeasy 96 Blood & Tissue Kit, Qiagen, Hilden, Germany). The sex of each sampled whale was determined by amplification of sex-specific markers following the protocols of Gilson et al.^56,57^. Results were compared to controls for a known male and female using gel electrophoresis.

Samples were genotyped using 10 previously published microsatellite loci to resolve the individual identity of each sampled whale and remove potential duplicates (Table 2)^58–62^. Alleles were sized and binned using the software program Genemapper v3.7 (Applied Biosystems). The total number of amplified loci for a given sample was considered as an added quality control threshold. Given the estimated probability of identity for these loci from previous studies^18,63^, we considered samples matching at a minimum of seven loci to be recaptures of the same individual. Samples with fewer than seven microsatellite loci were repeated or excluded. Recaptures of the same individual were removed from analysis. The expected probability of identity (P_ID_; the probability that two individuals are drawn at random from a population will have the same genotype by chance) for each locus was calculated in GenAlEx v6.5^64^. Cervus 3.0.7^65^ was used to compute the number of alleles (K), observed and expected heterozygosity (HO and HE), and the probability of identity for all individual matches.

**Table 2:** Summary of microsatellite loci used for individual identification of humpback whales (*Megaptera novaeangliae)* along the WAP^58–61^.

Locus	Source	Label	[mgCl_2_] mM	Size range (bp)	No. of alleles	H_E_	H_O_	P_ID_
Ev14	Valsecchi and Amos^58^	VIC	2.5	125–143	9	0.789	0.800	0.072
Ev37	Valsecchi and Amos^58^	NED	3.5	192–226	16	0.905	0.915	0.017
Ev96	Valsecchi and Amos^58^	FAM	1.5	143–173	13	0.870	0.875	0.030
GATA28	Palsbøll et al.^59^	NED	2.5	143–191	11	0.325	0.328	0.016
GATA417	Palsbøll et al.^59^	FAM	2.5	187–282	20	0.909	0.911	0.465
GT211	Palsbøll et al.^59^	FAM	2.5	100–120	10	0.818	0.82	0.056
GT23	Berube et al.^60^	VIC	2.5	101–123	9	0.728	0.728	0.113
GT575	Berube et al.^60^	FAM	1.5	137–177	14	0.814	0.836	0.056
rw4-10	Waldick et al.^61^	VIC	2.5	190–216	14	0.845	0.846	0.044
rw48	Waldick et al.^61^	NED	3	112–120	5	0.740	0.708	0.111

The number of alleles observed (H_O_) and expected (H_E_) heterozygosity) was calculated using *Cervus 3.0.1.* The expected probability of identity (P_ID_) of each locus was calculated with the program *GenAlEx v6.5*.

### Hormone extraction and quantification

We extracted steroid hormones from the blubber portion of the biopsy samples following standard methods^18,66^. Briefly, to quantify hormone biomarkers (i.e., progesterone and cortisol), a cross-sectional sub-sample (~ 0.15 g) spanning from the epidermis-blubber interface to the most internal layer of the biopsy was sub-sectioned. These sub-samples were then homogenized multiple times using an automated, multi-tube homogenizer (Omni International). Following the completion of the homogenization process, target hormones were isolated using a series of chemical washes, evaporations, and separations. The final hormone residue was stored at − 20° C until analysis. The amount of hormone in each extract was quantified using a commercially available enzyme immunoassay. The progesterone EIA kit (EIA kit 900–011, ENZO Life Sciences, Farmingdale, NY) that was utilized in this study has 100% reactivity with progesterone and an assay detection limit between 15 and 500 pg/mL. Two additional standard dilutions were added to allow for a lower detection limit of the standard curve to 3.81 pg/mL. The cortisol EIA kit (EIA kit K003-H1W, Arbor Assay, Ann Arbor, MI) that was used in this study has 100% reactivity with cortisol and an assay detection limit between 50 and 3200 pg/mL. Two additional standard dilutions were added to allow for a greater detection limit of the standard curve from 25 pg/mL to 6400 pg/mL. All samples were run blind and in duplicate for both hormones. For both assays, extracts were further diluted and re-run if reliable hormone concentrations were not obtained during the initial assay process. For cortisol, to avoid censoring the data, samples that fell below the detectable range of the assay were assigned a value half of the lowest standard curve (i.e. 12.5 pg/mL). Each assay was evaluated for color development using a Biotek plate reader Epoch (Gen5™ software [Biotek, USA]) with read and correction wavelengths of 405 nm and 630 nm for progesterone and 450 nm and 630 nm for cortisol. Blubber hormone concentrations were then transformed into nanograms of cortisol per gram of blubber (wet weight).

As part of our routine quality control, we determined the extraction efficiency by spiking subsamples of blubber from a stranded, dead humpback whale with the target hormone^66^. The percentage of hormone that was recovered after the extraction was calculated and each sample concentration was adjusted to this efficiency before statistical analyses. An extraction efficiency greater than 60% was acceptable. If the extraction efficiency was less than 60%, the sample extracts were discarded, and the blubber samples were re-extracted. Additionally, we conducted a parallelism test to gauge the performance of humpback blubber extracts with the cortisol EIA kit. This was done by taking a serially diluted pool of sample extracts and running them, along with the standard controls of the assay, to determine whether the linear decrease in measured values of the pooled sample was parallel to the standard curve. This would indicate that the assay measures the same antigens in the blubber as in the standards. Five extracts from four individual whales were pooled together (4-female; 2-biopsied, 2-stranded), and the pooled sample concentrations were made by diluting five times from the neat preparation to 1/32, decreasing by a factor of two. Each dilution was run two times, and the resulting curve of the concentrations as a function of the mean optical density was compared to the standard curve.

### Pregnancy classification

Pregnancy of female humpback whales was assigned following previously published methods^67^. Briefly, a series of biopsy samples (n = 29) were collected from individuals of a known life-history stage from the Gulf of Maine feeding aggregation by the Center for Coastal Studies in Provincetown, MA. Using these control samples from the Gulf of Maine, the pregnancy state relative to blubber progesterone concentrations was modeled using a standard logistic regression model^68^. Each WAP humpback sample, of unknown pregnancy status, was entered into the model and the model returned a probability of being pregnant for each female sampled^68^. If the probability of being pregnant was greater than 99%, that female was given a pregnant assignment. If the probability of being pregnant was less than 1%, that female was given a not-pregnant assignment. If a biopsied female's probability of being pregnant was between those two bounds, that female received an undetermined pregnancy assignment.

### Classification of life history groups

We classified individual females into four life history categories based on field observations during biopsy events and blubber progesterone concentrations. These included lactating females, pregnant females, lactating and pregnant females, and lastly resting females. Lactating females were those females that had a calf travelling near the female and whose behavior was in sync with the presumed mother (so although we do not know for certain these females were lactating, their behavior was consistent with females that are known to be lactating). Pregnant females were determined using progesterone concentrations as described above. Lactating and pregnant females were females that were pregnant based on progesterone concentrations and in proximity with a calf. Lastly, resting females included females that were not pregnant and or lactating, but could also have included females that recently lost a pregnancy.

### Vessel activity data

Vessel activity data for the Antarctic Peninsula region was obtained from the International Association of Antarctic Tour Operators (IAATO) statistics repository (https://iaato.org/information-resources/data-statistics/). IAATO has been carefully monitoring, analyzing, and reporting Antarctic tourism trends since 1991 as part of its commitment to the effective self-management of guest activities in the region. As part of these reports, IAATO generates seasonal statistics among the roughly 100 member vessels, including the number of operators, vessels, and voyages in each season, as well as the number of passengers visited. As part of the IAATO guidelines, member vessels are grouped into four categories: category 1 (12–200 passengers), category 2 (201–500 passengers), cruise only (501 + passengers), and yacht (12 passengers max). We used the total number of voyages across all categories as our explanatory metric to assess the relationship between humpback whale cortisol levels and human activity. This analysis does not account for the activity of vessels along the peninsula that are not IAATO members.

### Data preparation and statistical analyses

All statistical analyses were performed in R^69^. Cortisol concentrations were log-transformed to improve normality. We tested for differences in cortisol concentrations between sexes, pregnancy states, and across years of high and low vessel activity using a *Welch’s two sample t-test.* We used an ANOVA to test for differences in cortisol concentrations across the feeding season (Jan-Mar) and life history groups. We used a post-hoc Tukey's Honest Significant Difference (HSD) test to check for differences among individual explanatory variables (e.g., life history class). We used a general linear model to examine the relationship between ordinal date and vessel activity on cortisol concentrations. Unless otherwise specified, all cortisol values are reported as mean ± standard error (ng/g wet weight). For all statistical tests, we considered a *p*-value of less than 0.05 to be significant. Sampling periods for this study were bounded to the months of January–March of 2019–2021 when sampling was most uniform across the three years.

### Animal ethics

All research protocols were evaluated and approved under scientific research permits issued by the National Marine Fisheries Service (NMFS) under the authority of the Marine Mammal Protection Act of 1972 (permit numbers: 14809 and 23095). The National Science Foundation (NSF) Antarctic Conservation Act (ACA) permits (2015–011 and 2020–016) were obtained to conduct biopsy sampling of baleen whales in the Antarctic Peninsula Region. Our study is reported in accordance with ARRIVE guidelines and UC Santa Cruz’s Institutional Animal Care and Use Committee (IACUC) approved protocols for the collection of whale biopsy samples (UCSC permits Friea1706 and Friea2004). Additional sample collection was conducted under the Ministry of Education and Science of Ukraine Permit Series AP № 075–19/2. The samples originating from outside the US jurisdiction were imported under the Convention on International Trade in Endangered Species (CITES) import/intro from the sea permit numbers 19US504849/B, and 20US60410D/9-21US60410D/9. Lastly, all experiments were performed in accordance with relevant guidelines and regulations.

## Supplementary Information

Supplementary Information.

## Data Availability

The datasets supporting this article have been uploaded as part of the electronic supplementary material.
